# Adaptive Proteome Diversification by Nonsynonymous A-to-I RNA Editing in Coleoid Cephalopods

**DOI:** 10.1093/molbev/msab154

**Published:** 2021-05-22

**Authors:** Yoav Shoshan, Noa Liscovitch-Brauer, Joshua J C Rosenthal, Eli Eisenberg

**Affiliations:** 1 Raymond and Beverly Sackler School of Physics and Astronomy, Tel Aviv University, Tel Aviv, Israel; 2 The Eugene Bell Center, Marine Biological Laboratory, Woods Hole, MA, USA

**Keywords:** RNA editing, adaptation, evolution

## Abstract

RNA editing by the ADAR enzymes converts selected adenosines into inosines, biological mimics for guanosines. By doing so, it alters protein-coding sequences, resulting in novel protein products that diversify the proteome beyond its genomic blueprint. Recoding is exceptionally abundant in the neural tissues of coleoid cephalopods (octopuses, squids, and cuttlefishes), with an over-representation of nonsynonymous edits suggesting positive selection. However, the extent to which proteome diversification by recoding provides an adaptive advantage is not known. It was recently suggested that the role of evolutionarily conserved edits is to compensate for harmful genomic substitutions, and that there is no added value in having an editable codon as compared with a restoration of the preferred genomic allele. Here, we show that this hypothesis fails to explain the evolutionary dynamics of recoding sites in coleoids. Instead, our results indicate that a large fraction of the shared, strongly recoded, sites in coleoids have been selected for proteome diversification, meaning that the fitness of an editable A is higher than an uneditable A or a genomically encoded G.

## Introduction

Adenosine-to-inosine (A-to-I) RNA editing (Bass 2002; Nishikura 2010; Eisenberg and Levanon 2018), catalyzed by enzymes of the ADAR family (Savva et al. 2012), is probably the most common RNA modification in metazoa. The vast majority of RNA editing activity occurs in noncoding regions of the transcriptome, with millions of editing sites found in noncoding regions of the genome in humans and other species (Bazak et al. 2014; Ramaswami and Li 2014; Picardi, D’Erchia, et al. 2017; Porath, Knisbacher, et al. 2017). However, some of the edits do reside within protein-coding RNA sequences. As inosines are usually interpreted by the translation machinery as guanosines (Basilio et al. 1962; Licht et al. 2019), edits may result in amino-acid substitutions (a process termed “recoding”) and modify the function of the protein product. Recoding has the capacity to create a range of proteins isoforms from a single gene, providing the organism with a new mechanism for acclimation and adaptation. Editing levels at specific sites may be programmed in a slow process of evolutionary adaptation, whereby cis mutations affect the dsRNA structures that are recognized by ADAR, thus controlling the editing efficiency of the target. In most cases, editing is not complete and the edited and unedited versions of the protein are expressed concurrently. Moreover, editing levels may vary even between neighboring cells (Harjanto et al. 2016; Picardi, Horner, et al. 2017). Thus, editing is fundamentally different than a genomic mutation in that it allows for expressing multiple protein isoforms, with varying levels of each, in response to environmental changes (Rieder et al. 2015). Thus, one may expect that in the course of organism’s evolution, recoding sites will appear and be fixed in the transcriptome as a means for adaptation and acclimation to variable external pressures (Gommans et al. 2009; Rosenthal 2015).

Although the adaptive potential of editing is obvious, the extent to which it is used remains unclear. In mammals, thousands of recoding (nonsynonymous, *N*) events have been recorded in public databases, mostly in humans (Ramaswami and Li 2014; Picardi, D’Erchia, et al. 2017). The vast majority of these are not conserved across mammals and, based on several criteria, do not show signs of selection (Xu and Zhang 2014). First, they are depleted and more weakly edited than synonymous (*S*) sites. Moreover, they are under-represented in essential genes, highly expressed genes, and genes that are under purifying selection. These observations suggest that most nonconserved mammalian recoding sites are likely to be functionally unimportant, nonadaptive, and neutral or slightly deleterious. However, these conclusions may be affected by the low precision of current compilations of mammalian recoding sites (Tan et al. 2017).

A few dozen recoding sites are known to be conserved across mammals (Pinto et al. 2014). These sites tend to be more strongly edited, and exhibit a higher ratio of recoding to synonymous sites (*N*/*S* ratio), indicating positive selection (Xu and Zhang 2015). Interestingly, with the exception of the essential Q/R site within *GRIA2* transcripts (Higuchi et al. 2000), complete abolishment of recoding is well tolerated in mice (Chalk et al. 2019). Thus, with a single exception, the phenotype produced by recoding in conserved mammalian targets is expected to be subtle (Horsch et al. 2011) or apparent only under specific conditions. Functional studies have been published for only a small number of physiologically important mammalian recoding targets (Sommer et al. 1991; Egebjerg and Heinemann 1993; Lomeli et al. 1994; Burns et al. 1997; Sailer et al. 1999; Bhalla et al. 2004; Yeo et al. 2010; Daniel et al. 2011; Huang et al. 2012; Bazzazi et al. 2013; Chen et al. 2013), and the implications of recoding to the cell and the organism remain largely unknown for the vast majority of reported sites. It is therefore yet unknown whether the few studied examples indicate that conserved recoding sites in mammals generally alter functional properties in precise ways, or, in many cases, are also nonadaptive.

Editing by ADARs is common to all multicellular metazoa. However, the repertoire of recoding sites varies considerably across lineages (Pinto et al. 2014; Alon et al. 2015; Duan et al. 2017; Liscovitch-Brauer et al. 2017; Zhang et al. 2017; Porath et al. 2019; Duan et al. 2021) with only a single edit known to be shared between mammals, insects, and cephalopods (Porath et al. 2019). Notably, editing is highly abundant in coding sequences of the coleoid cephalopods, where tens of thousands of sites have been identified, mostly in neural tissues (Albertin et al. 2015; Alon et al. 2015; Liscovitch-Brauer et al. 2017). The functional impact of recoding has been demonstrated for several targets in cephalopods (Patton et al. 1997; Rosenthal and Bezanilla 2002; Colina et al. 2010; Liscovitch-Brauer et al. 2017), but it is generally unknown for thousands of conserved, strongly edited, recoding sites, and so far adaptation through recoding has not been demonstrated on a large scale.

Strongly edited cephalopod sites do exhibit a high *N*/*S* ratio (Alon et al. 2015; Liscovitch-Brauer et al. 2017), which is widely considered as an indication of positive selection and an overall beneficial effect of recoding. More accurately, one looks at *f*_N_, the rate of nonsynonymous sites, defined as the fraction of the number of *N* sites to the number of potential genomic adenosines whose editing would have resulted in a nonsynonymous change. Similarly, *f*_S_ is the rate of synonymous sites. The ratio of these two rates (*f*_N_/*f*_S_; see Materials and Methods) exceeding unity is a signature of positive selection. In addition, other signatures for positive selection of editing have been recently reported (Moldovan et al. 2020; Popitsch et al. 2020). However, the applicability of these measures as a signature for adaptation in the context of recoding was recently challenged (Jiang and Zhang 2019; Moldovan et al. 2020; Popitsch et al. 2020). It was pointed out that over-representation of nonsynonymous edits may be explained by fixation of recoding events compensating for harmful G-to-A mutations. These A-to-I recoding events are indeed beneficial in the sense they restore the damage caused by the G > A genomic mutation (“harm-permitting editing”) (Jiang and Zhang 2019). Similarly, recoding may be beneficial as it introduces inosines in positions where a genomic A > G mutation would be beneficial, regardless of the ancestral state (Moldovan et al. 2020; Popitsch et al. 2020). In these cases, although recoding may be functionally important and evolutionary maintained, there is no adaptive advantage to having an editable A as compared with a genomically encoded G. Therefore, positive selection of recoding does not prove that the flexibility in having both edited and nonedited isoforms is, in itself, adaptive. It is still possible that recoding is maintained by evolution even if only the protein version encoded by the edited transcript is advantageous.

The “harm-permitting” model is supported by a previous analysis of cephalopod recoding sites which showed an enrichment of recoding in restorative sites (sites for which the edited version of the transcript encodes for an ancestral version of the protein) (Jiang and Zhang 2019), in accordance with prior studies in other species (Tian et al. 2008; Zhang et al. 2014; An et al. 2019). Further, it was suggested that “diversifying editing,” where recoding introduces an amino-acid that was not present ancestrally, is suppressed (*f*_N_/*f*_S_*<* 1), indicating an overall deleterious effect. Finally, as expected by the harm-permitting model (HPM), editing efficiency is higher at restorative sites. It was thus suggested that there is no evidence for an adaptive advantage due to the ability to produce two products from the same genomic locus, and virtually all recoding sites are either deleterious (A allele is better) or harm permitting (G allele is better) (Jiang and Zhang 2019).

In this study, we present an alternative explanation for the analyses that were used to support the HPM and show that they are also consistent with an adaptive model. We then show how the evolutionary dynamics of recoding sites provides a more stringent test for the harm permitting hypothesis. Assuming no adaptive advantage for recoding beyond introduction of a preferred genomic G allele (as expected according to the HPM), there should be no selective pressure against mutating these recoded adenosines to genomically encoded Gs. One can then obtain a conservative estimate for the minimum number of A > G genomic substitutions expected to evolve at recoding sites. Comparing this lower bound to the actual numbers observed, we find a highly significant suppression of these mutations, suggesting that a sizable fraction of strongly edited recoding events in coleoids are adaptive in the strong sense, namely it is beneficial to have an editable A at these positions, over both uneditable A and genomically encoded G.

## Results

### Two Competing Hypotheses: The HPM and the Adaptive Model

**Fig. 1.**
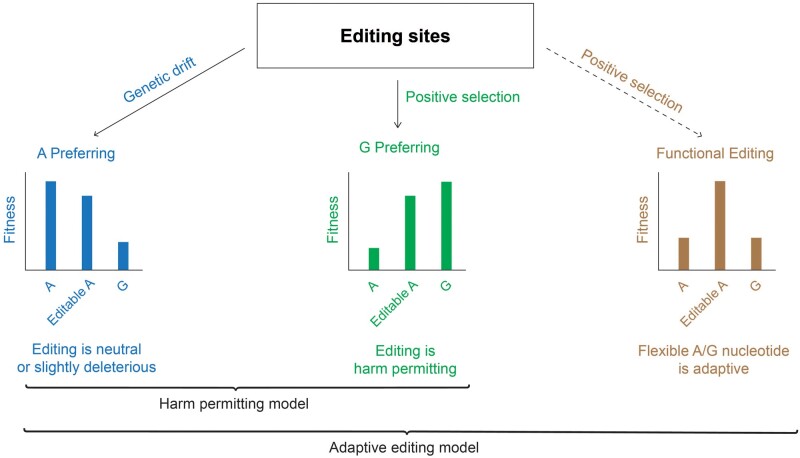
The harm-permitting and adaptive editing models. Recoding sites may be fixated into the genome due to random genomic drift, even though their editing does not provide any selective advantage and may be even slightly deleterious. These randomly fixated sites (A-preferring sites) are not expected to be enriched in nonsynonymous editing (left). In a second class of recoding sites, editing does provide a selective advantage as it replaces an inferior A allele by the preferred G allele (middle). For these G-preferring sites, editing does increase the fitness of the organism over an uneditable A, but having a genomically encoded G is equally beneficial or even better (middle). In both cases, fitness does not depend on the protein diversity and flexibility provided by recoding. The HPM asserts that all (or almost all) recoding sites belong to these two categories. In contrast, according to the adaptive editing model some of the recoding sites belong to a third class, where having a recodable codon is functionally important, and the editable A provides a selective advantage over both an uneditable A and a genomically encoded G (right).

For the sake of clarity, we first define explicitly the two competing hypotheses which we would like to compare (fig. 1). For each recoding site, one should consider the overall fitness of three possible alternatives: an uneditable genomic A, a genomic G, and the observed editable A. The adaptive editing model (AEM), specifically, postulates that for an appreciable subset of recoding sites, the editable codon is adaptive, as it provides higher fitness by increasing proteome diversity and flexibility.

Alternatively, the HPM asserts that the diversification of the proteome achieved by recoding does not provide a selective advantage. That is, for each recoding site there exists a single version of the protein (either edited or unedited) that would provide a higher overall fitness than having the two versions produced by editing. This must hold true across all environmental conditions, tissues and developmental stages, etc. Accordingly, all recoding sites can be divided into two classes. The first is the A-preferring sites, where recoding is either neutral or weakly deleterious. Recoding at these sites provides no selective advantage, and the fitness of the organisms would be equal or higher if recoding were abolished altogether. The second is the G-preferring sites, where recoding does provide a selective advantage by compensating for a less fit genomic A. Recoding at these sites is neutral or slightly deleterious compared with a genomically fixed G. In summary, according to HPM virtually all recoding sites are either A-preferring or G-preferring.

Note that these two competing hypotheses may be described in terms of four recently described hypotheses concerning the adaptive nature of editing events, named H1 to H4 (Popitsch et al. 2020). HPM assumes all editing sites are either slightly deleterious (H2) or compensate for a harmful A allele (H3/H4). In contrast, AEM assumes that in addition to these classifications, some of the sites are adaptive due to the increased diversity of the transcript population (H1). In contrast to a recent analysis (Popitsch et al. 2020) which looked for a single hypothesis that best explains the observed population-genomics signatures, here we consider HPM and AEM which allow for the possibility of different sites behaving differently (Jiang and Zhang 2019; Moldovan et al. 2020).

### Constructing the Editome for Six Coleoid Species

**Fig. 2.**
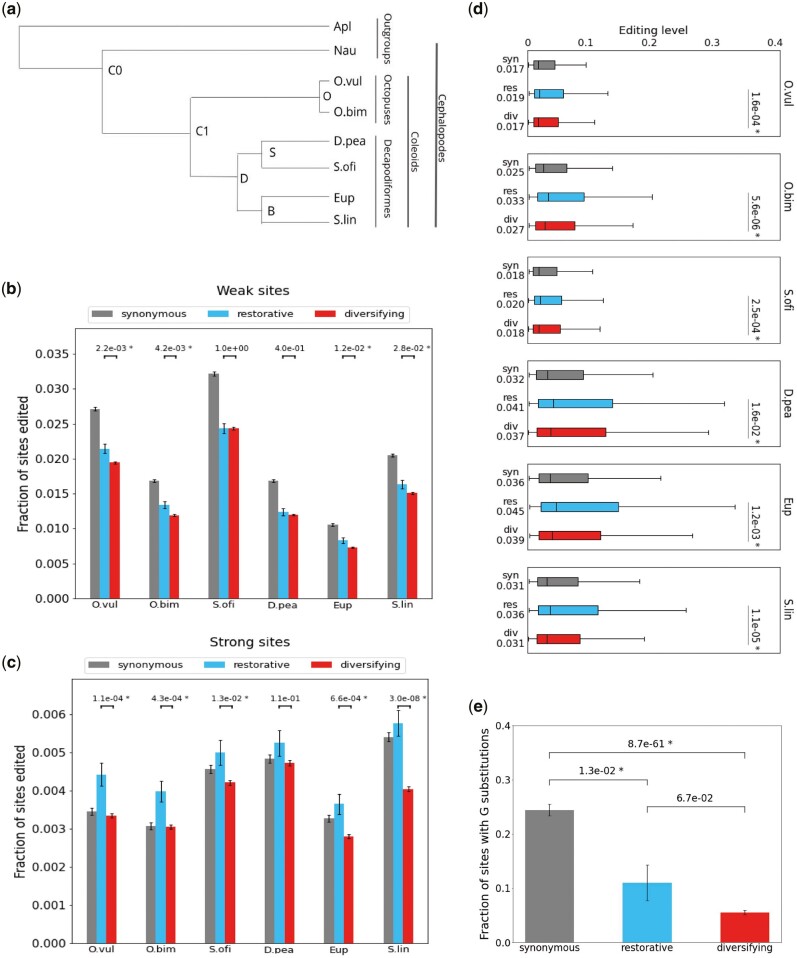
Restorative and diversifying editing. (*a*) Phylogeny of the eight species studied here: six coleoids and two outgroup mollusks (see Materials and Methods). (*b*) Incidence of weak editing (<10%) in synonymous, restorative, and diversifying sites, for each of the six coleoid species. *P* values for the difference between the incidence rate of restorative and diversifying sites are indicated (Fisher’s exact test). (*c*) Same as (*b*), for strong (>10%) editing sites. (*d*) Box plots showing the distribution of editing levels for synonymous, restorative, and diversifying sites, per species. The boxes represent the first-to-third quartiles range, the horizontal line within the box indicates the median, and the whiskers extend to the most extreme values within a window sized 1.5 times the box size, centered at the median. *P* values for the difference between the editing levels of restorative and diversifying sites are indicated (Mann–Whitney test). (*e*) Fraction of sites edited in C1 (LCA of the six coleoids studied) that were mutated into a genomic G in at least one of the six descendant species. Significance of difference for each pair of groups is indicated (Fisher’s exact test). *P* values < 0.05 are marked with an asterisk. Error bars represent SEM.

To test the HPM, we looked at the evolution of editing sites across the coleoid lineage. A’s at G-preferring sites are expected to be replaced by genomically fixed G’s at a rate that is greater than or equal to the neutral mutation rate. As explained below, this leads to a quantitative prediction (or a lower bound) for the total number of G > A mutations in ancestral editing sites, which we will compare with the actual number of mutations observed. We analyzed the neural transcriptomes of eight mollusk species (fig. 2*a*). Six of these species are coleoids that exhibit extensive recoding in neural tissues. These include the previously analyzed (Liscovitch-Brauer et al. 2017) *Octopus vulgaris*, *Octopus bimaculoides*, *Doryteuthis pealeii* (longfin inshore squid), and *Sepia officianalis* (cuttlefish), to which we have now added *Euprymna scolopes* (Hawaiian bobtail squid) and *Sepioloidea lineolata* (striped pajama squid) (see supplementary table S1, Supplementary Material online for sequencing details). In addition, we analyzed two evolutionary outgroups that were studied previously (Liscovitch-Brauer et al. 2017) and do not exhibit extensive recoding, a nautiloid (*Nautilus pompilius*; a noncoleoid cephalopod) and a gastropod mollusk (*Aplysia californica*). The transcriptomes of these species were constructed de novo (supplementary table S2, Supplementary Material online), and editing sites were identified for the six coleoid species as described previously (Alon et al. 2015; Liscovitch-Brauer et al. 2017) (Materials and Methods and supplementary table S3, Supplementary Material online). We have identified 4,557 gene groups for which an orthologous gene was found in each of the six coleoid species, harboring 206,531 unique editing sites (a site edited in multiple species is counted once; supplementary table S4, Supplementary Material online). In addition, we have identified 2,613 gene groups for the eight molluscan species (including nautilus and aplysia), in which 128,700 editing sites reside (supplementary table S5, Supplementary Material online). Some of the editing sites are detected in only one species, whereas others are shared by multiple species. Note that the distribution of codon changes due to editing is similar for all six coleoid species (supplementary fig. S1 and table S6, Supplementary Material online), and the codon usage shows some differences between coleoids and the other two species analyzed (supplementary table S7, Supplementary Material online). We then determined the ancestral genomic nucleotide for each editing site and, if it is an adenosine, its ancestral editing status (see Materials and Methods). This data set (supplementary tables S4 and S5, Supplementary Material online) is used below to test the HPM.

Despite multiple morphological and molecular analyses, the exact phylogeny of Decabrachia is still not well resolved (Tanner et al. 2017; Sanchez et al. 2018; Anderson and Lindgren 2021). Thus, we used the abovementioned 2,613 (molluscan) gene groups to reconstruct the phylogeny of the eight species studied here (fig. 2*a*, Materials and Methods). Below we also consider alternative topologies of the phylogenetic tree that appear in the literature, and show that our main results are robust to the differences between the alternative phylogenetic trees.

### Restorative Editing Is More Abundant and Stronger

It was pointed out previously (Jiang and Zhang 2019) that both the frequency and the recoding levels are higher for restorative sites (nonsynonymous editing sites where the amino acid encoded by the edited transcript has appeared in one of the ancestral versions of the protein; see Materials and Methods) compared with nonrestorative sites, in accordance with the HPM. We find similar results in our extended analysis. For both weak (<10%) and strong (>10% editing) editing sites, the incidence in restorative sites is higher than nonrestorative sites (fig. 2*b* and *c*). For strong editing sites, the incidence of restorative sites is even higher or equal to that of synonymous strong sites. Furthermore, editing levels are also higher in restorative sites (fig. 2*d*). These results are consistent with the possibility that the excess of nonsynonymous sites may be explained by restorative, harm-permitting, editing (Jiang and Zhang 2019) or by editing at positions where the A allele is less preferred than the G allele (Moldovan et al. 2020; Popitsch et al. 2020).

However, these results are also consistent with the AEM. A necessary (but not sufficient) prerequisite for adaptive editing, where fitness increases by the flexibility to produce both unedited and edited alleles, is that both alleles are tolerated, regardless of whether one of the two alleles is more beneficial. Clearly, restorative sites, where a genomic G was demonstrated to be tolerated in one of the ancestral species, are more likely to tolerate editing. Therefore, even according to AEM it is not surprising to find a higher incidence of recoding in these sites compared with random adenosines. Put differently, the results of figure 2*b–d* show that restorative sites are more likely to become strongly edited, but they do not tell us whether this editing is merely harm permitting. These sites could very well be utilized to diversify the proteome.

### Restorative Editing Is Not Necessarily Harm Permitting

In order to examine further the two possibilities presented by the HPM and the AEM, we looked at the fraction of ancestral editing sites (sites edited in C1, the LCA of the six coleoids studied here, see fig. 2*a*) that were mutated into G in at least one of the descendant species (fig. 2*e*). These sites, where editing has been conserved for 200–350 My (Kröger et al. 2011), are likely to be enriched in functional editing. According to HPM, their editing should be directed at restoring a preferred G allele, and thus we expect the nonsynonymous A > G mutation rate at these sites to be at least as high as the neutral mutation rate (estimated by the mutation rate observed for synonymous sites). In contrast to this expectation, we find that highly conserved, nonsynonymously edited A’s are mutated to G much less frequently than those at synonymous sites. This result holds regardless of whether the ancestral amino acid at C0 was identical to the edited version (restorative) or to the unedited one (diversifying). In fact, the mutation rate for restorative sites is not significantly different than for diversifying sites (p=0.07). These data suggest that at least some of the highly conserved, ancestral, recoding sites are not merely harm permitting and the genomic G allele is selected against, regardless of their being diversifying or restorative. Clearly, finding an ancestral version of the protein identical to the one encoded by the edited version of the transcript is not sufficient to label a site as G-preferring and to assume its only function is to restore the ancestral genomic allele. On the other hand, a diversifying site may in principle be G-preferring, if the recoded amino acid is preferred by the descendant species.

### Using the Genomic Mutation Rate to Test the HPM

The data presented in figure 2*e* cast doubt on the use of the ancestral allele to classify sites as A-preferring or G-preferring and calls for further scrutiny of HPM. Here we present an approach for testing HPM against the AEM based on the evolution of editing sites along the coleoid lineage, without assuming whether a specific site is A- or G-preferring.

HPM asserts that all recoding sites are A-preferring or G-preferring. The A-preferring sites are neutral or slightly deleterious and were presumably fixed in the genome due to random mutations and genetic drift. Accordingly, one may expect the fixation of novel A-preferring editing sites to occur at a rate not higher than that of synonymous editing sites (probably lower, as there might be a detrimental effect to recoding if the A allele is actually superior).

Furthermore, as these sites prefer a genomic A, A > G mutations at these sites should be suppressed (compared with neutral synonymous mutations). The A > G mutation rate at these sites is expected to be similar to that of general nonsynonymous A > G mutations, or maybe somewhat higher (as these sites are already known to tolerate G to some extent). 

**Fig. 3.**
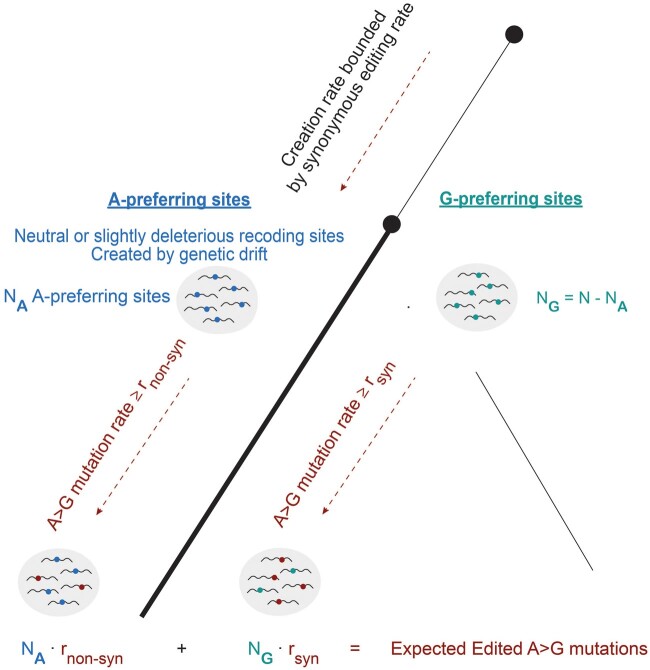
The number of expected A > G mutations of ancestrally strongly edited sites based on the HPM. According to HPM, all ancestral strong editing sites are either A-preferring or G-preferring. A-preferring sites are neutral or slightly deleterious, and thus an upper bound to their incidence rate at the ancestral node (lower black circle) is obtained from the incidence rate of synonymous sites at the same node. The remaining sites are G-preferring (a lower bound). Mutations are expected to accumulate along the evolutionary path from the above ancestral node to each of the descendants (thick black line). A-preferring sites are expected to mutate along this path at a rate higher than the background nonsynonymous mutation rate, and G-preferring sites are expected to mutate at a rate higher than the neutral (synonymous) rate. Together, one obtains a conservative estimate to the number of mutations expected based on HPM, to be compared with the observed numbers.

In coleoid cephalopods, however, strongly edited nonsynonymous sites are actually enriched and are found at a higher rate than synonymous ones. The HPM attributes this excess to G-preferring sites, whose recoding is beneficial as a substitution for an A > G mutation. For this class of sites, there should be no selective pressure suppressing genomic A > G mutations. The rate of such mutations should be equal or higher to the neutral mutation rate, which is the rate observed for synonymous A > G mutations. As we detail below (see fig. 3) these considerations lead to a specific prediction for the expected number of A > G mutations to occur within a set of ancestral strong editing sites (editing level >10%).

### The Expected Number of Mutations according to the HPM

For a given a set of *N* nonsynonymous and *S* synonymous sites that were strongly edited in a common ancestor, HPM asserts that all *N* nonsynonymous sites can be classified to two groups: *N*_A_ strongly edited A-preferring sites and *N*_G_ strongly edited G-preferring sites, where *N* = *N*_A_ + *N*_G_. Although we have no direct way of identifying to which category a specific site belongs, we can still estimate the size of the two groups (*N*_A_ and *N*_G_), as follows.

As explained above, the fixation of novel A-preferring strongly edited editing sites is equal or lower than the fixation rate for evolutionary-neutral strong sites, which can be estimated by the rate for synonymous strong editing sites. The latter is given by $S/A_{S}$, where S is the number of strong synonymous sites and $A_{S}$ is the total number of synonymous adenosines in the ancestral genome (genomic adenosines whose substitution into G does not change the encoded amino acid). However, A-preferring sites are expected to be slightly deleterious (due to the fraction of transcripts harboring the nonpreferred G allele), and thus to be suppressed by natural selection and be found at a lower rate. This suppression is indeed observed even for weak (<5% edited) sites (supplementary fig. S2, Supplementary Material online), which are likely all A-preferring (Liscovitch-Brauer et al. 2017), as the observed ratio between nonsynonymous and synonymous rates of weak editing sites is lower than unity. Quantitatively, this ratio is given by the multiplicative depletion factor $d_{W}=\;\left(N_{W}/A_{N}\right)\;/\;\left(S_{W}/A_{S}\right)$ where $N_{W},S_{W}$ are the number of weak nonsynonymous and synonymous sites, respectively; $A_{N}$ is the total number of nonsynonymous genomic adenosines. We argue that the suppression of A-preferring nonsynonymous sites should be even more pronounced for strongly edited sites, where the detrimental effect of editing is larger due to the higher editing levels. Thus, we use the depletion factor calculated for weak sites as a lower bound for the actual depletion factor that applies to the strong A-preferring sites. This leads to the following upper bound for the actual number of strongly-edited A-preferring sites, from which we obtain a lower bound for the number of strongly-edited G-preferring sites:

$$N_{A}\leq \;\frac{S}{A_{S}}A_{N}d_{W}\;N_{G}\;\geq \;N-\;\frac{S}{A_{S}}A_{N}d_{W}.$$

Using these bounds, one can now estimate the expected number of A > G mutations to be found in a descendant species at ancestrally edited positions. Ancestral G-preferring sites are expected to be mutated to G at a rate equal to or higher than the neutral rate, *R*_S_, which can be estimated by the observed rate of A > G mutations at all synonymous sites (edited or not edited). On the other hand, the mutation rate of ancestral A-preferring sites is at least as large as the nonsynonymous A > G mutation rate, *R*_N_ (probably larger, as these sites accommodate weak editing, so having a G there is likely less harmful than for the typical nonsynonymous A). Adding these numbers, we obtain a lower bound for the expected number of A > G mutations at the ancestral strong recoding sites (fig. 3).

### Genomic Mutations Are Suppressed at Strong, Shared, Recoding Sites

We have performed the above calculations with the ancestral species being either 1) S, the LCA of *D. pealeii* and *S. officianalis* or 2) B, the LCA of *E. scolopes* and *S. lineolata* or 3) D, the LCA of all these four species. For each of these, we compared the number of expected and observed mutations accumulated between the ancestor and each of its descendants (eight different evolutionary paths in total). The evolutionary paths branching from the LCA of the two octopus species are too short for meaningful comparisons. Notably, in all eight comparisons the observed number of mutations are far less than the expected numbers (supplementary table S8, Supplementary Material online), suggesting that strongly edited sites resist A > G mutations, in contrast with the predictions of HPM.

**Fig. 4.**
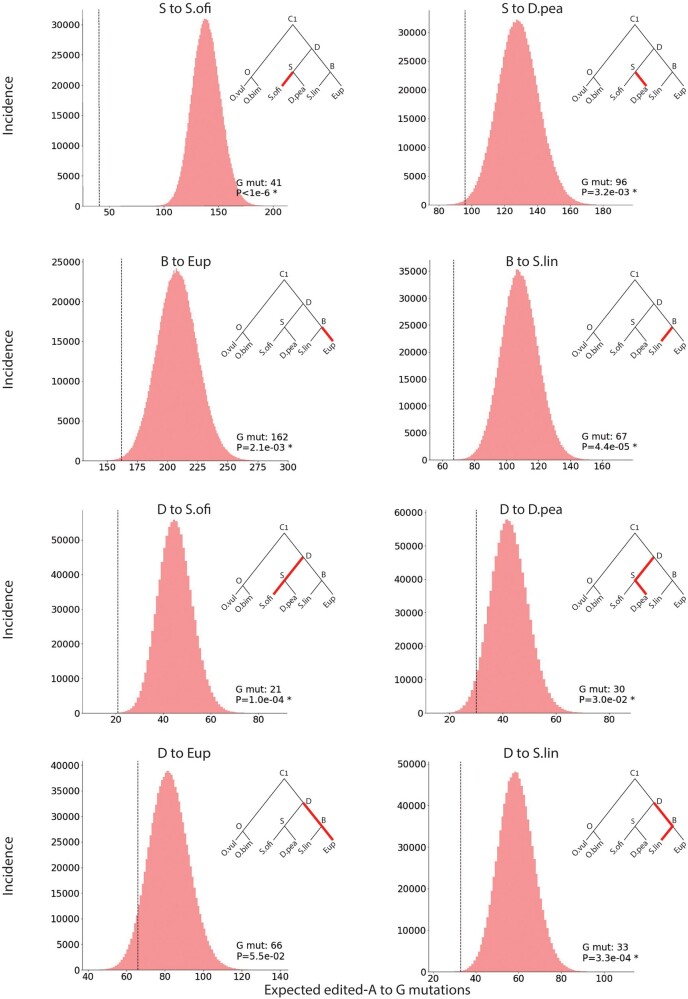
A > G mutations are depleted at strong editing sites. Panels Compare the actual numbers of A > G mutations in ancestral strongly edited sites (vertical dashed line, numbers indicated in legend) with the distributions based on HPM assumptions and background transcriptomic data. Note that the eight paths (and thus the eight statistical tests) are not independent. Distributions plotted based on 10^6^ samples of the statistical model. Vertical axis represents the number of instances generated in the above process within each bin range. *P* values calculated by direct comparison with the distribution. *P* values < 0.05 are marked with an asterisk.

Even if all of the rates above were precisely known, one would expect a random distribution of the actual number of mutations around the mean. Furthermore, the estimates for the rates introduce further stochastic fluctuations. Thus, to quantify the statistical significance of the differences presented in supplementary table S8, Supplementary Material online, we have applied a statistical model (Materials and Methods) to obtain the full distribution for the expected number of mutations, given HPM assumptions and the available data. Using these distributions, one can assign *P*-values to each of the observed number of mutations assuming the null-hypothesis HPM (fig. 4). These results strongly support the AEM.

In order to obtain a quantitative estimate for the fraction of adaptive sites, we have extended our model as follows: we now postulate that a certain (predetermined) fraction of the strong nonsynonymous sites is adaptive. To be stringent, the adaptive sites are assumed to be perfectly maintained by evolution and thus do not contribute to the number of A > G mutations. We therefore apply our HPM-based model to the rest of the sites, as previously described. This results in a distribution for the number of A > G mutations, which depends on the value of the additional parameter (the fraction of adaptive strong nonsynonymous sites). Within this conservative model, our best estimator for the fraction of adaptive sites is the value of the parameter for which the actual number of A > G mutations observed equals the median of the distribution. Using this approach, we obtain estimates for the fraction of all strongly edited sites that are recoding-preferring (rather than A-preferring or G-preferring), ranging from 11% to 41% for the eight evolutionary paths analyzed (see supplementary table S8, Supplementary Material online for confidence intervals).

Note that by the nature of the analysis, our estimate applies only to sites shared by at least two of the species. Furthermore, one cannot tell the total number of recoding-preferring sites since we do not know which sites are recoding-preferring for each path examined and the overlap between the numbers found for different paths is also not known. Finally, due to the bounds used throughout our estimates, the numbers above underestimate the actual fraction of recoding-preferring sites.

Recoding activity in cephalopods is higher in neural tissues (Alon et al. 2015; Liscovitch-Brauer et al. 2017); we therefore checked whether neural-specific genes exhibit a different behavior compared with widely expressed genes. We call a gene neural-specific if its expression level averaged over four neural tissues is four-fold larger than the level averaged over eight non-neural tissues, based on *O. bimaculoides* data (Albertin et al. 2015) (see Materials and Methods). As expected, the overall incidence rate of editing is indeed ∼2-fold higher for neural genes. Furthermore, although for non-neural genes non-restorative recoding is somewhat suppressed compared with synonymous editing (*f*_N_/*f*_S_ < 1), in neural genes non-restorative recoding is enriched or (in the single case of *S. lineolata*) significantly less suppressed (supplementary fig. S3, Supplementary Material online). Separate analysis of A > G mutations in recoding sites within neural-specific genes does not yield consistent and significant results, due to the reduced statistical power in this smaller set of recoding sites.

In order to verify the robustness of our results, we have verified that they are insensitive to the details of the phylogenetic tree, or to the parameters used to define weak and strong sites. Since there are some uncertainties regarding the exact phylogeny of Decapodiformes, we have verified that our conclusions are essentially unchanged when using two alternative evolutionary trees (supplementary figs. S4–S7 and tables S9 and S10, Supplementary Material online). In addition, we have reanalyzed the data using several choices for the cutoff values defining weak and strong sites (supplementary table S11, Supplementary Material online). Stricter cut-offs (e.g., >20% editing for strong sites or <1% editing for weak sites) results in smaller sets of sites and suppress the statistical power. However, for all combinations of parameters one observes a clear suppression of A > G mutations as compared with HPM predictions. Finally, we considered the possibility that the mutations rates are slower in edited genes. Therefore, we repeated the analysis using synonymous and nonsynonymous rates calculated based on edited genes only. Since conserved editing is associated with a slower mutation rate in the vicinity of the editing sites (Liscovitch-Brauer et al. 2017), we estimated the mutation rates based on adenosines that are at >200 bp apart from any detected nonsynonymous editing site. The results are presented in supplementary figure S8 and table S12, Supplementary Material online. For all evolutionary paths examined, the number of observed mutations is still lower than expected, and even though the associated *P*-values are higher, the results are significant for most paths.

## Discussion

Recent decades have revealed the important role played by post-transcriptional and post-translational mechanisms in generating the proteomic complexity of higher organisms. Recoding by A-to-I RNA editing is one such mechanism, well positioned to facilitate proteome diversification. It may create a range of proteins from a single gene in a temporally regulated, tissue-specific, condition-dependent manner and provide a new means for acclimation and adaptation. Indeed, tantalizing hints for recoding being exploited to respond to external conditions, circadian rhythm and sleep have been emerging (Garrett and Rosenthal 2012; Robinson et al. 2016; Porath, Schaffer, et al. 2017; Terajima et al. 2017; Yablonovitch et al. 2017). On the other hand, there are very few cases where the utility of producing both the edited and unedited versions of a protein has been convincingly demonstrated.

Restorative, harm-permitting editing is likely to be an important function of recoding. The first discovered example of A-to-I recoding—the Q/R site in mammalian *GRIA2* transcripts—is plausibly a fine demonstration of this function. However, we provide evidence that this is not the only way in which recoding is utilized, and a selective advantage for recoding over both an unedited adenosine and a genomically fixed guanosine can be demonstrated for a sizable fraction of positively selected recoding sites in the neural transcriptome of coleoids.

Differentiating between the HPM and AEM evolutionary models is not easy and requires a large statistical sample. Here we used the uniquely large editome of the coleoid cephalopods to make the case for adaptive recoding. Further analyses are required to examine the question whether this finding can be extended to other taxa, including mammals. Additionally, computational methods for identifying the specific sites where proteomic plasticity plays a functional role are desired. Then, the functional changes caused by these editing events can be assessed with activity assays. Finally, genetically tractable cephalopod models will allow us to directly compare the effects of editable versus uneditable positions on fitness. These approaches will help reveal the adaptive role played by transcriptomic plasticity and will promote our understanding of this powerful epigenetic mechanism.

## Materials and Methods

### Subject Details

Previously described RNA and DNA sequencing data were analyzed for six of the species: *Doryteuthis pealeii* (giant fiber lobe [GFL] and optic lobe [OL]; PRJNA255916) (Alon et al. 2015); *Octopus bimaculoides* (OL, supraesophageal ganglia [supra], suboesophageal, and the axial nerve cord; PRJNA270931, PRJNA285380) (Albertin et al. 2015); *Aplysia californica* (ten different tissues; PRJNA13635, PRJNA77701); *Octopus vulgaris* and *Sepia officinalis* (SG and OL); and *Nautilus pompilius* (supra and OL; PRJNA300723) (Liscovitch-Brauer et al. 2017).

In addition, we produced and analyzed data for *Sepioloidea lineolata* and *Euprymna scolopes*. *Sepioloidea lineolata* specimens were captured off the coast of Sydney, Australia. Animals were cultured for four generations at the Marine Biological Laboratory by the Cephalopod Initiative. *Euprymna scolopes* founder specimens were captured at the Kewalo Marine Lab, Oahu, Hawaii and cultured for two generations at the Marine Biological Laboratory by the Cephalopod Initiative. A single individual of each species was selected for DNA and RNA extraction. Samples destined for RNA extraction were immersed in chilled, filtered, seawater, and immediately preserved in RNAlater. Gills samples intended for DNA extraction were flash frozen in liquid nitrogen. All samples were then stored at −80 °C. RNA from SG and OL was extracted with the RNAqueous kit (Life Technologies, Carlsbad, California), and genomic DNA was extracted from the gills using Genomic Tip Columns (QIAGEN, Venlo, Limburg, The Netherlands).

Genomic DNA sequencing libraries were prepared using the TruSeq DNA Sample Prep kit, as described by the manufacturer (Illumina, San Diego, California), and sequenced using three lanes of the Illumina HiSeq 2000 instrument, with paired-end, 101 nt reads. RNA-seq libraries (*Euprymna scolopes*: OL and SG; *Sepioloidea lineolata*: right and left OL, SG, skin and white body) were prepared using the TruSeq Stranded mRNA Sample Prep Kit, as described by the manufacturer (Illumina), and each sample was sequenced using one lane of Illumina HiSeq 2000 instrument, with 151 nt reads. The number of reads generated for each tissue is presented in supplementary table S1, Supplementary Material online.

#### Transcriptome Assembly and Editing Detection

Transcriptome assembly and editing site detection were done as described in previous works (Alon et al. 2015; Liscovitch-Brauer et al. 2017). In short, de novo transcriptome for each species was assembled from RNA reads of all abovementioned samples using Trinity (Grabherr et al. 2011) (version Trinity-r2012-10-05). Putative open-reading frames (ORFs) were inferred by similarity to known proteins (Blastx *E*-value < 1e-6) in the Swiss-prot proteins data sets (Bateman et al. 2017). To construct the editome, DNA and neural RNA reads were then separately aligned to the assembled transcriptomes using Bowtie2 with local alignment configurations and default parameters (Langmead and Salzberg 2012) in order to detect editing events in each tissue. For each species, the editome used for further analysis is the unification of editomes for all neural tissues of that species (excluding nonCNS samples). For more information regarding transcriptome assembly and editome detection, see Alon et al. (2015) and Liscovitch-Brauer et al. (2017).

For each putative protein-coding sequence, the region found to be similar to a Swiss-prot protein was extended in both directions until a stop codon or the end of a component was reached. In some cases, the resulting region includes stop codons (found within the similarity region), in which case we assumed the longest ORF to be the coding sequence. However, if two adjacent ORFs were separated by stop codons which overlap a stop-loss editing event (UAG → UGG editing, STOP → W), we considered them as a single long ORF, as the edited transcript may translate into a longer protein version. We maintained only ORFs which are at least 50 amino-acid long. Details of the generated transcriptomes are presented in supplementary table S2, Supplementary Material online. Statistics of the editomes analyzed appear in supplementary table S3, Supplementary Material online.

#### Ortholog Grouping and Multiple Sequence Alignment

For each pair of animals, we used Orthomcl (Li et al. 2003) to identify orthologous genes, using only best two-way hits and default parameters. Considering a group of animals (either all eight species described in this text, or the six coleoids studied), we define orthologous groups as sets of genes (one gene per species) that are pairwise orthologous for each pair in the group. Ortholog genes were translated to protein sequences (using W, tryptophan, in cases of editable stop codons), and aligned using Clustal-Omega (Sievers et al. 2011) with default parameters to obtain multiple sequence alignment (MSA) for each orthologous group. The protein MSA was then converted back to nucleotide MSA using pal2nal (Suyama et al. 2006).

#### Phylogenetic Analysis

To construct a phylogenetic tree for the eight species studied, we concatenated all 2,613 orthologous groups for the eight species into a single MSA, and applied RaxML-NG (Kozlov et al. 2019) (version-1.0.1) with default parameters (and two seeds for the pseudo-random number generator to ensure reproducibility). The MSA was analyzed in three independent ways: amino-acids MSA was analyzed using either WAG or LG substitution model, and the nucleotides MSA was analyzed using the GTR+Gamma substitution model. These three independent analyses yielded 60 different trees, all of which showing the exact same topology (RF distance = 0). We also used RaxML bootstrap convergence assessment option to verify that bootstrapping test converges quickly (after 50 trees replicates, with RaxML autoMRE bootstrapping criteria [–bs-cutoff] ≤ 1%). We then used RaxML to generate 50 bootstrap trees for each of the three models used to infer the topology, taking all 150 trees to generate support values for the inner branches of the inferred topology. All inner branches were fully supported (100/100).

Our analyses in the main text apply to this tree topology (fig. 2*a*), rooted at the branch leading to the outgroup *Aplysia*. This tree is consistent with Anderson and Lindgren (2021) and figure 1 of Sanchez et al. (2018). However, despite the clear results of the above analysis, in light of the uncertainty in the Decabrachia phylogeny (Tanner et al. 2017; Sanchez et al. 2018; Anderson and Lindgren 2021), we have also analyzed the data using two other trees (see supplementary figs. S4–S7, Supplementary Material online) to ensure our results are robust to the details of the tree.

#### Constructing the Ancestral Sequences

Ancestral sequences were reconstructed using the codeml program of the PAML4 package (Yang 2007) with default parameters and the topology of the evolutionary tree. Our analysis does not use the lengths of the tree branches, and thus we used a rooted tree while executing codeml.

#### Analysis of Editing Sites Types according to HPM

To calculate the rates of synonymous/restorative/diversifying sites (fig. 2*b* and *c*) and their editing levels (fig. 2*d*) we have followed the methodology of Jiang and Zhang (2019). A nonsynonymous editing site was considered restorative if the amino acid encoded by the edited version of the transcript is found to be genomically encoded in the reconstructed genomic sequence for one of the ancestral species, up to and including C0 (see fig. 2*a*), and diversifying otherwise. The rates of synonymous/restorative/diversifying editing sites are just the number of such sites divided by the number of all the adenosines (in all gene groups analyzed) that would have been synonymous/restorative/diversifying if edited.

To calculate A > G substitutions rates in conserved sites (fig. 2*e*), we analyzed all sites deemed to be edited at C1, the ancestral species of all coleoids (i.e., sites edited in at least one current species of decapodiformes and at least one current species of octopodes, see below). These sites were classified to synonymous and nonsynonymous based on the effect of editing to the ancestral sequence, and to weak (<10%) or strong (>10%) sites based on the average editing level over all current coleoids species edited at the site. Finally, we have classified these conserved ancestral C1-edited sites as restorative if the sequence of the C0 ancestor (the LCA of all cephalopods, fig. 2*a*) encodes the amino acid of the edited C1 transcript and diversifying otherwise. The substitution rate is the fraction of sites for which the ancestral genomic A was mutated into a genomic G in at least one of the current coleoid species.

#### Deriving the HPM Distributions for the Number of Expected Mutations

The MSAs include lowly conserved regions, or regions which are conserved (or well-assembled by Trinity) only in some of the species while presenting long gaps in others. These regions are prone to errors in determining the ancestral sequence. Thus, we limited the following analyses to positions (MSA columns) for which at least 30% of the amino acids within a window ranging ten amino acids upstream and downstream are fully conserved, taking into account (for each of the evolutionary paths considered in fig. 4) all current species but the one for which we estimate the number of mutations. In other words, for each nucleotide and for each species, we looked at the amino-acids MSA surrounding the codon that includes this nucleotide, and excluding the species being analyzed. We focused on a window of 21 amino acids surrounding the codon that includes the analyzed nucleotide (21 columns, for the amino acid considered, 10 upstream and 10 downstream amino acids). The nucleotide was discarded unless there are at least 30% of the 21 positions for which the amino acid found in all five species is identical to the consensus. We have verified that our results are not sensitive to these window parameters.

### Inferring Editing in Ancestral Species

As editing sites are a small fraction of all adenosines and our editing detection pipeline is prone to miss many true editing sites, an adenosine was considered to be edited in an ancestral species if it was detected as edited in at least two species for which this ancestor is the LCA. In addition, we assumed the site to be edited if it was edited in an ancestral species and in a descendant species. For example, for a site observed to be edited in *D. pealeii* and in *O. bimaculoides* but no other current species, we infer editing in the LCA of these (which we call C1, the ancestral coleoid). We further deduce that descendants of C1 which are ancestors of *D. pealeii* were also edited (e.g., D, the ancestor of all Decapodiformes). However, editing may have been lost along evolutionary paths leading to current species for which editing is not observed, for example the LCA of *Sepioloidea lineolate* and *Euprymna scolopes*.

For current species, the editing level per site per species was calculated by pooling the RNA-seq data from all neural tissues considered for this species (see above). For ancestral editing sites, we estimate the editing level as the average of editing levels observed in all current species that exhibit editing at this site.

Clearly, it is more difficult to infer ancestral editing at a site that was mutated to G in one current species, due to the lower amount of data (only information from unmutated species can be used for this inference). To avoid this selection bias, for each evolutionary path being tested we inferred the ancestral editing state and the ancestral editing levels using only the information from species that do not belong to this path.

### Mutation and Editing Rates

The rates of synonymous/nonsynonymous A > G genomic mutations from an ancestral species to a current species, and the rates of synonymous/nonsynonymous A-to-I editing sites events created at a given ancestral species were calculated as the ratio of such events to the number of synonymous/nonsynonymous adenosines that exist in the ancestral species. An adenosine is considered synonymous if mutating it into a G does not change the encoded amino acid, and nonsynonymous otherwise.

We call editing in an ancestral species only if it was detected as edited in at least two species that does not belong to the path examined, at least one of which is a descendant of the ancestral species considered. Therefore, our edited adenosine sites may be more conserved than the typical adenosine. To compensate for that, we calculate the mutation rates of (N or S) unedited sites for similarly conserved adenosines, considering only ancestral adenosines which are maintained as adenosines in at least two species that do not belong to the path examined, at least one of which is a descendant species.

### Evaluating the Distribution of the Number of Mutations under HPM

As explained in the main text, HPM assumptions lead to an estimate of the expected number of nonsynonymous A > G mutations in a given set of editing sites along a specific evolutionary path. However, these estimates are only averages, and statistical sampling noise may lead to different numbers. We therefore treat the number of ancestrally edited sites for which a specific descendant species would exhibit a guanosine as a statistical random variable, and evaluated its distribution according to HPM assumptions. This distribution is then compared with the actual number of mutations observed.

To construct the distribution, we describe the number of mutations in terms of (a convolution of) simple random processes for which the distribution is known. We then randomly sample all these distributions to obtain the number of mutations in a specific realization, as detailed below. Repeating this process multiple times, one obtains the full distribution (Devroye 2006).

(A) Discrete events, such as single synonymous/nonsynonymous mutation or creation of a single editing site with specific characteristics (synonymous or nonsynonymous; certain range of editing level) are described as binary variables with a probability p to occur. The total numbers of such events, X, are therefore binomially distributed: X ∼ B(p,N) with *N* being the number of relevant adenosines in the data set at which this event may have happened (e.g., for synonymous site creation or a synonymous mutation, *N* is the number of all As that are synonymous with respect to an A > G substitution). The maximum-likelihood estimator for p is thus $\hat{p}=\;\frac{x}{N}$ (while x is the number of events that are observed in the data set among the N relevant adenosines).

However, the actual rate may deviate from the best estimator. We therefore sample the “true” rate, for each of the above events from a normal distribution $r∼G\left(\hat{p},\sqrt{\hat{p}\left(1-\hat{p}\right)/N}\right)$, where $\hat{p}$ is the above maximum-likelihood estimator (the Gaussian distributions were truncated at 0 and 1 as values outside this range are meaningless). This procedure is applied in each realization, to find the rates for creation of weak (editing level <5%) and strong (editing level >10%), synonymous and nonsynonymous editing sites, as well as the rates for synonymous/nonsynonymous A to G mutations.

(B) As explained in the main text, the expected number of strongly edited A-preferring editing sites is (at most) $\frac{S}{A_{S}}A_{N}d_{W}$, where S is the number of strong synonymous sites, $A_{N}\;\mathrm{and}\;A_{S}$ are the total number of nonsynonymous/synonymous adenosines in the ancestral genome, and $d_{W}$ is the depletion factor $d_{W}=\left(N_{W}/A_{N}\right)/\left(S_{W}/A_{S}\right)$. Put it differently, the expected number of strongly edited A-preferring editing sites is (at most)

$$\lambda_{A\;\mathrm{pref}.}=\frac{\mathrm{Rate}\;\mathrm{of}\;\mathrm{weak}\;\mathrm{nonsyn}\;\mathrm{sites}}{\mathrm{Rate}\;\mathrm{of}\;\mathrm{weak}\;\mathrm{syn}\;\mathrm{sites}}\cdot \;\mathrm{Rate}\;\mathrm{of}\;\mathrm{strong}\;\mathrm{syn}\;\mathrm{sites}\;\cdot A_{N}$$

Here too we replace the observed rates by the randomly sampled rates (as explained above), to get a value of $\lambda_{A\;\mathrm{pref}.}$ for each specific realization. Since the number of reconstructed adenosines in each of the ancestral species (internal nodes) is large (>$10^{6})$, and the events at distantly located sites are largely independent, the random number describing the actual number of strong A-preferring sites, A pref, may be assumed to be a Poisson variable with a mean of $\lambda_{A\;\mathrm{pref}.}$ and the number of strong nonsynonymous G preferring sites is then

G pref. =Total sites − A pref.

(C) The actual number of nonsynonymous A to G mutations in A/G-preferring sites in this realization is then sampled from two Poisson distributions, with means

$$\lambda_{G\;\mathrm{mutations}\;\mathrm{in}\;A\;\mathrm{pref}.\;}=A\;\mathrm{pref}.\;*\;r_{\mathrm{nonsyn}\;\mathrm{AG}\;\mathrm{mutations}},$$

$$\lambda_{G\;\mathrm{mutations}\;\mathrm{in}\;G\;\mathrm{pref}.}=G\;\mathrm{pref}.\;*r_{\mathrm{syn}\;\mathrm{AG}\;\mathrm{mutations}}.$$

Adding these two numbers one obtains the total number of nonsynonymous A > G mutations in ancestral strong editing sites for this specific realization.

For each evolutionary path examined in this work, we have repeated the above process $10^{6}$ times to obtain the distribution for the number of A > G mutations in strong nonsynonymous sites accumulated along this path. We then counted how many of these $10^{6}$ realizations resulted in a number of mutations that is equal or lower than the actual number of A > G mutations observed. The fraction of such cases gives the significance (*P*-value) of the result.

#### Estimating the Fraction of Adaptive Sites

In order to estimate the proportion of adaptive sites (sites in which an editable A is presumably preferable), we have extended the above model as follows. We assumed a certain fraction of strong nonsynonymous sites is adaptive, and subtracted the adaptive sites from the total count of strong nonsynonymous sites. We assume, stringently, that the adaptive A sites are never mutated to G, so that the expected A > G mutations all come from the nonadaptive sites. We therefore applied our HPM-based model using the numbers of nonadaptive sites to get the distribution of the number of expected A > G mutations. We used the bisection method to find the value of the adaptive fraction for which the median of the distribution equals the number of observed mutations. This value is considered to be our best estimate for the fraction of adaptive sites under the described model (the actual number is likely larger, as the model is conservative and under-estimates the number of expected mutations). In addition, we used the bisection method to find parameter values for which the probabilities to obtain a number of mutations exceeding the observed one are 0.025 or 0.975. These values define the 95% confidence interval for the adaptive fraction.

#### Classification of Neural and Nonneural Genes

To calculate the rates of restorative/diversifying/synonymous editing sites in neural and nonneural genes separately, we have applied SALMON (Patro et al. 2017) (version 0.11.2) with default parameters to map RNA-seq samples from 12 body tissues of *O. bimaculoides* to its Trinity-assembled transcriptome. Transcripts for which the relative abundance (averaged over the four neural tissues) exceeds 1 transcript per million (TPM) and is at least 4-fold higher than the average level in the eight nonneural tissues were classified as neural transcripts. Transcripts of all the other seven species were classified as neural/non-neural according to the status of their *O. bimaculoides* orthologue. To avoid possible editing-detection bias, we compared the neural transcripts with an expression-matched subset of non-neural genes that was generated as follows: For each neural gene, we picked (without replacement) the closest non-neural gene (in terms of TPM value). The median value for the ratio of TPM values for the matched pairs was 0.04%, and a difference of >10% was found in only two neural genes.

## Supplementary Material

Supplementary data are available at *Molecular Biology and Evolution* online.

## Supplementary Material

msab154_Supplementary_Data
