# Supplementary material for: Adaptive Proteome Diversification by Nonsynonymous A-to-I RNA Editing in Coleoid Cephalopods
Source: Mol Biol Evol. 2021 May 22;38(9):3775–88. doi: 10.1093/molbev/msab154 (PMC8382921; doi:10.1093/molbev/msab154)
Supplement: msab154_Supplementary_Data [file msab154_supplementary_data.zip › Supplementary Material_20210502.docx]

## Supplementary Figures


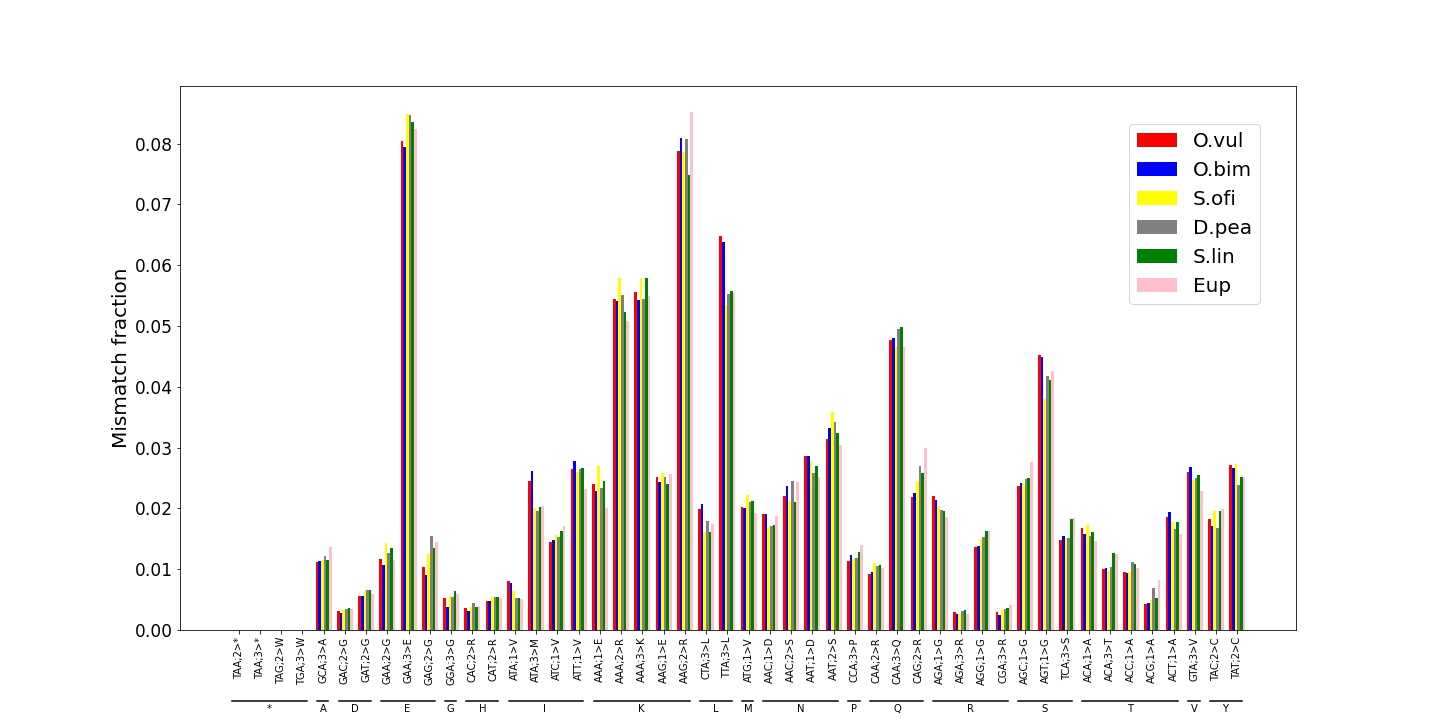


Figure S1: Distribution of editing events by codons (see also Table S6). Positions of edited adenosines in codons is indicated by the numbers adjacent to the codons labels on the horizontal axis. The distribution of edited codons is similar for the six coleoid species, and mostly follows the one expected based on codon usage and the local ADAR motif (see Alon et al, 2015).


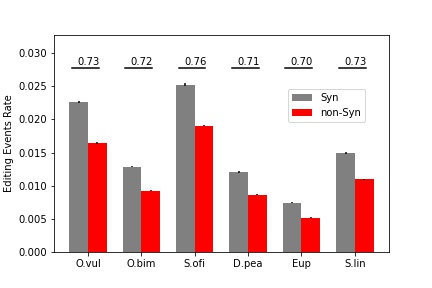


Figure S2: **Depletion of non-synonymous editing for weakly edited sites.** The incidence of non-synonymous weak editing is 0.7-0.76 times lower than that of synonymous weak editing, reflecting the slightly deleterious nature of weak non-synonymous editing. The suppression of strongly edited A-preferring sites is probably even more pronounced, but our model assumes, conservatively, that the same depletion factor applies for strongly edited A-preferring sites.


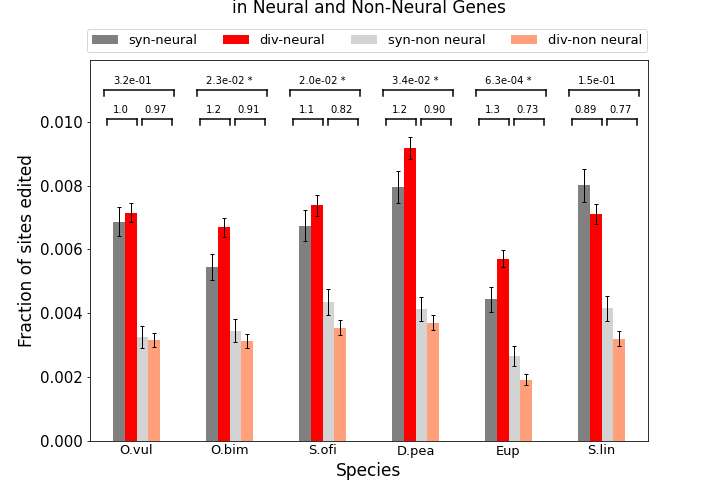
Figure S3: **Non-restorative recoding in neural genes is enriched.** Incidence rates of strong editing (>10%) in synonymous and diversifying sites for each of the six coleoid species, for neural and non-neural genes. Neural genes are defined as genes for which the average expression level in four neural tissues is four-fold larger than that in eight non-neural tissues, based on *O. bimaculoides* data^39^. All other genes are called non-neural. For each species, the ratio (*f*_N_ */f*_S_) between synonymous and diversifying non-synonymous sites is presented for neural and non-neural genes, separately (numbers in lower row), as well as P-values measuring the significance of the difference between the two odds ratios (two-sample z-test for ln(OR); upper row). Error bars represent s.e.m. P-values<0.05 are marked with an asterisk.


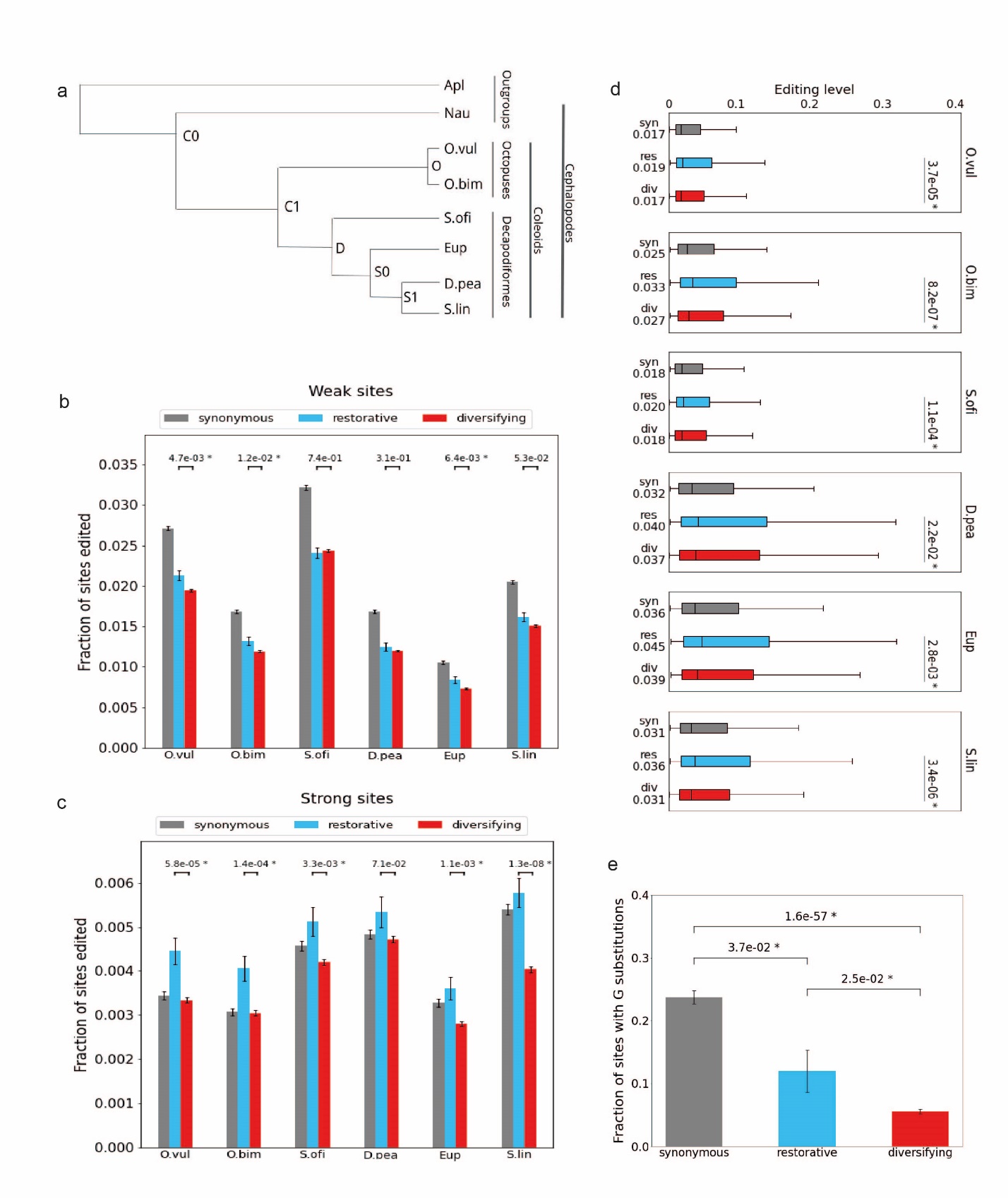


Figure S4: **Restorative and diversifying editing – first alternative evolutionary tree topology**. (a) An alternative evolutionary tree for the species considered, suggested by Dr. Oleg Simakov (private communication), exhibiting a topology different from the one considered in the main text (for the Decapodiformes species) and consistent with Tanner et al (2017). (b-e) Same we figure 2, for the alternative tree.


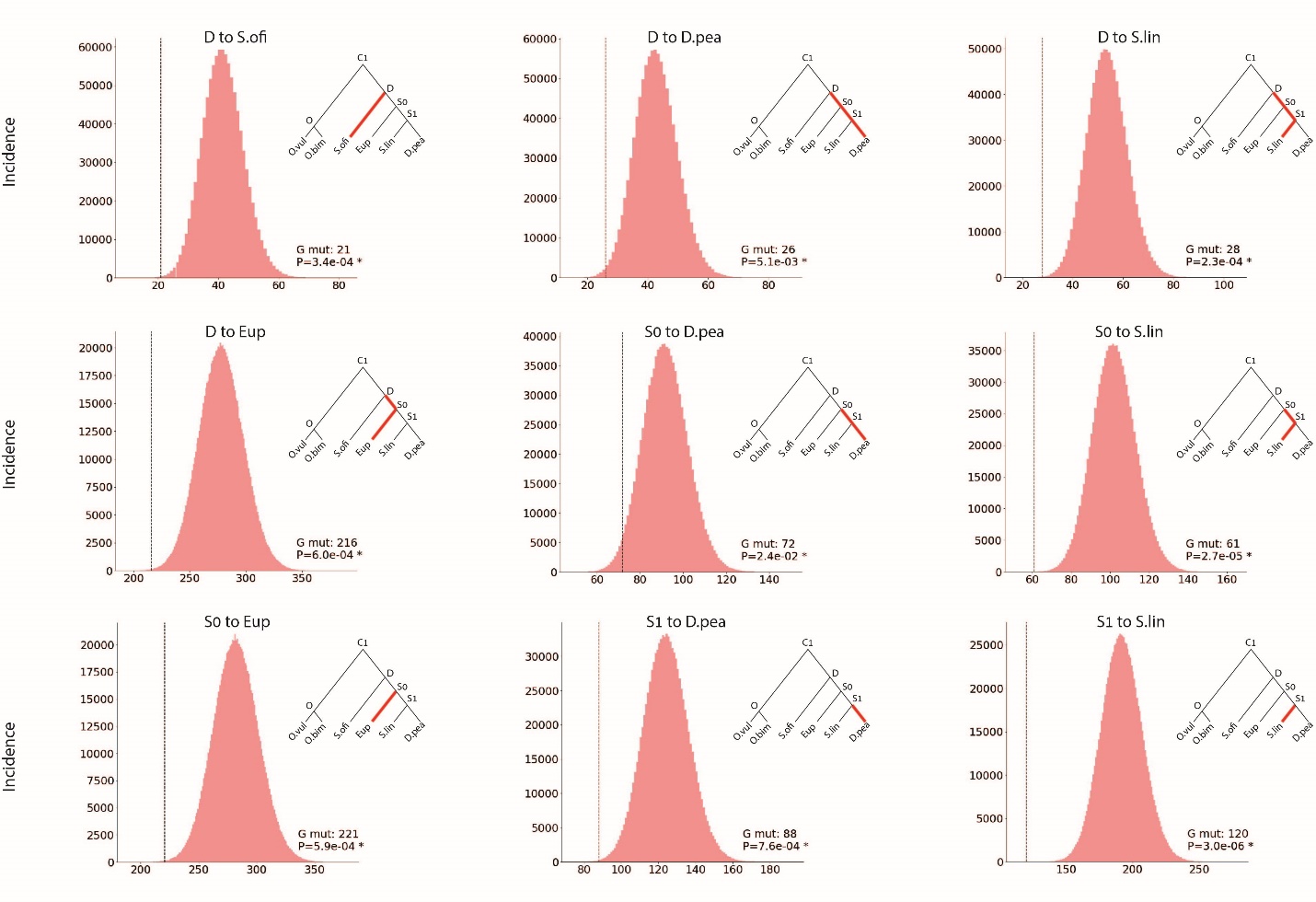


Figure S5: **A>G mutations and distributions of expected A>G mutations at strong editing sites – first alternative evolutionary tree topology**. Same as Figure 4, for the first alternative evolutionary tree (see Fig. S4). In this case, we examine 9 evolutionary paths. For all paths, the number of A>G mutations observed in strong ancestral sites is significantly smaller than the one expected by HPM


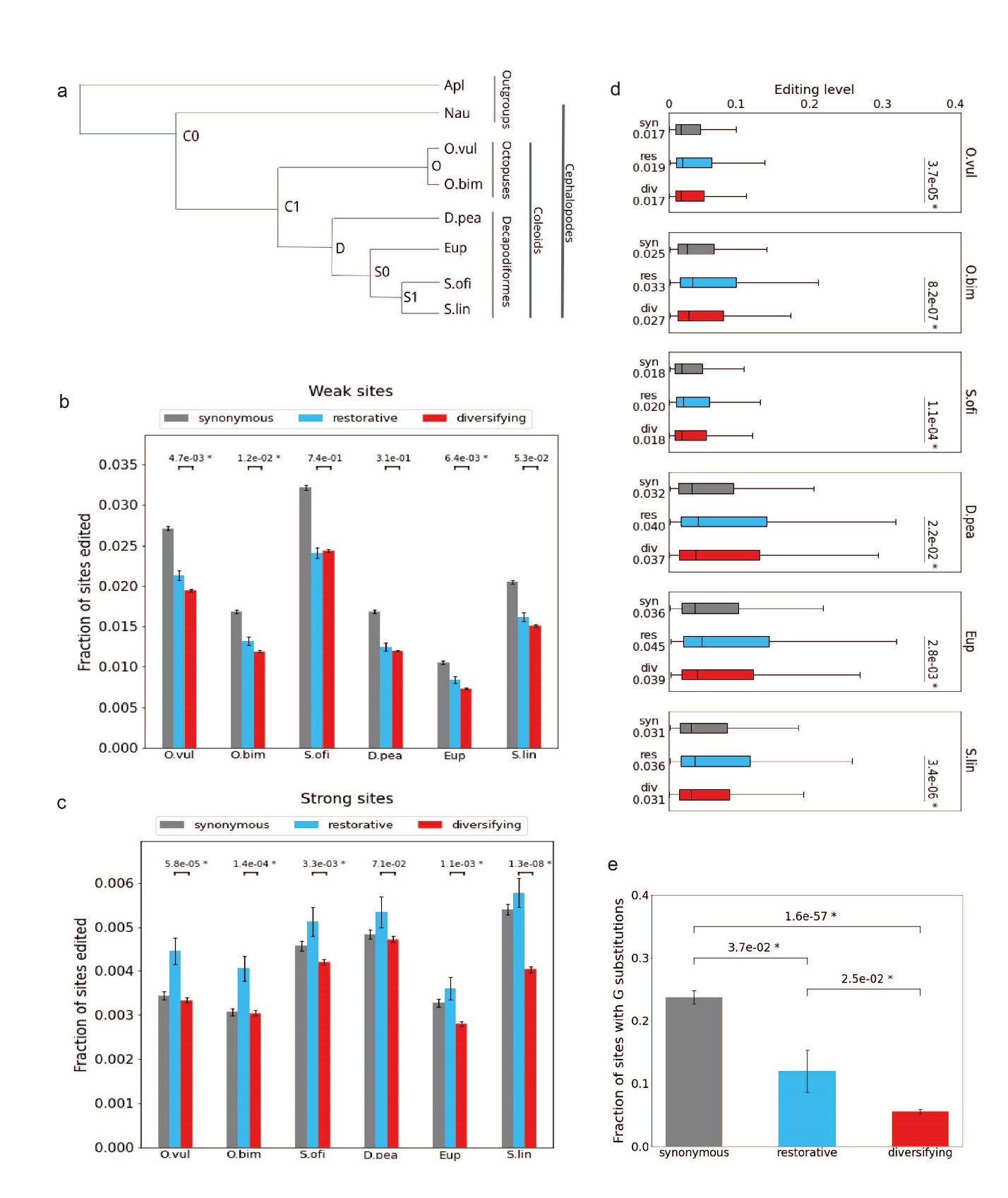


Figure S6: **Restorative and diversifying editing – second alternative evolutionary tree topology**. (a) An alternative evolutionary tree for the species considered, based on NCBI taxonomy browser, exhibiting a topology different from the one considered in the main text for the Decapodiformes species. (b-e) Same we figure 2, for the second alternative tree.


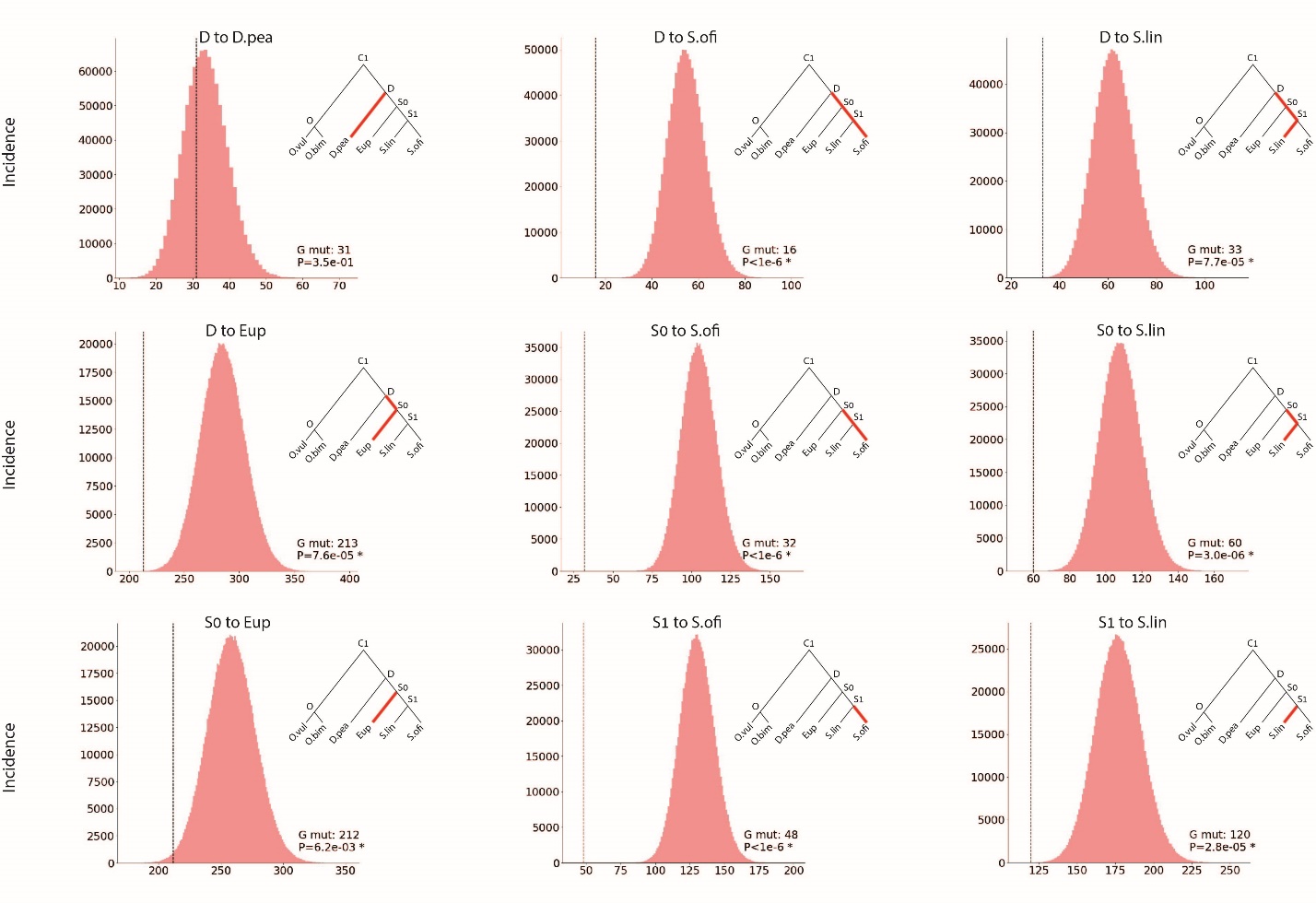


Figure S7: **A>G mutations and distributions of expected A>G mutations at strong editing sites – second alternative evolutionary tree**. Same as Figure 4, for the second alternative evolutionary tree based on NCBI taxonomy browser (see Fig. S6). In this case, we examine 9 evolutionary paths. For all paths, except the one from the ancestral node D to D. pealeii, the number of A>G mutations observed in strong ancestral sites is significantly smaller than the one expected by HPM.


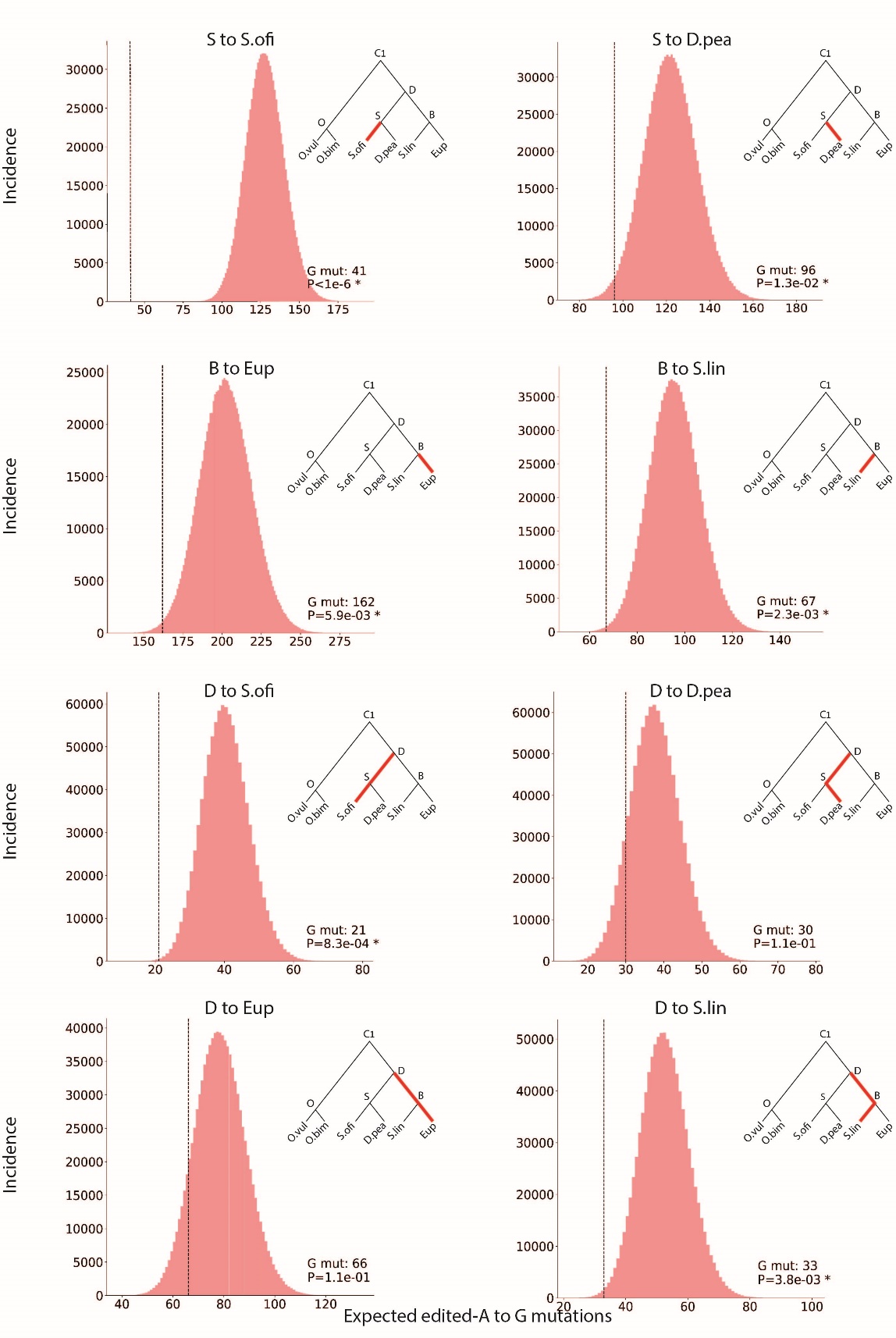


Figure S8: **A>G mutations and distributions of expected A>G mutations at strong editing sites, using mutations rates calculated from edited genes only**. Same as Figure 4, using mutation rates calculated based on nucleotides within edited genes, and at least 200bp apart from known editing sites (see Table S12). For six of the eight paths, the number of A>G mutations observed in strong ancestral sites is significantly smaller than the one expected by HPM.

## Supplementary Tables

**Tables are supplied as separate Excel files (S1-S3, S6) or gzipped tab-separated text (S4,S5)**

**Table S1**: Details of DNA and RNA sequencing data, for *Sepioloidea lineolate* and *Euprymna scolopes*.

**Table S2**: Statistics of the de-novo constructed transcriptome for the eight species.

**Table S3**: Statistics of the editomes for the six coleoid species.

**Table S4**: Editing sites analyzed (six species MSA)

**Table S5**: Editing sites analyzed (eight species MSA)

**Table S6**: Distribution of codon changes due to editing.

**Table S7**: Codons usage for the eight species examined.

**Table S8-S10**: Estimating the expected number of mutations under HPM, and a comparison to the actual numbers observed, for all 3 evolutionary trees examined.

**Table S11**: Estimating the expected number of mutations under HPM, and a comparison to the actual numbers observed, using our evolutionary tree and for different strong/weak editing levels cutoffs.

**Table S12:** Comparison of the observed mutation rates for all genes and for edited genes only.

**Table S13:** Estimating the expected number of mutations under HPM, and a comparison to the actual numbers observed, using our evolutionary tree and mutation rates estimated from edited genes only.

Please note: the large tables S4 and S5 are available at <http://www.tau.ac.il/∼elieis/squid>.
